# INTERVENTIONS TO IMPROVE GAIT SPEED

**DOI:** 10.1093/geroni/igae098.1916

**Published:** 2024-12-31

**Authors:** Jennifer Brach, Valerie Shuman, Subashan Perera

**Affiliations:** University of Pittsburgh, Pittsburgh, Pennsylvania, United States; University of Pittsburgh, Pittsburgh, Pennsylvania, United States; University of Pittsburgh, Pittsburgh, Pennsylvania, United States

## Abstract

Numerous evidence-based interventions exist to treat patients’ mobility limitations once identified in primary care. We will present an overview, focusing on the Program to Improve Mobility in Aging (PRIMA) study as one example. Interventions to improve walking historically focused on underlying impairments, with modest improvements. We hypothesize mobility limitations are a result of the loss of motor skill in walking and timing and coordination exercises targeting the skill of walking are an essential component of a mobility intervention. Participants >64 years were randomized to the standard strength and endurance (STD, n=125) or standard-plus timing and coordination (PLUS, n=124) intervention, 50-60 minutes, twice a week for 12 weeks. Participants with slower initial gait speed (< 1.0m/s) in both groups improved gait speed after the intervention (STD: 0.111m/s, PLUS: 0.088m/s) and maintained those improvements at the 12-week follow-up (STD: 0.087m/s, PLUS: 0.086m/s). In contrast, participants who initially walked faster (≥1.0m/s) in both groups improved gait speed after the intervention (STD: 0.060m/s, PLUS: 0.074m/s) but only those in the PLUS intervention maintained those improvements at the 12-week follow-up (STD: 0.027m/s, PLUS: 0.052m/s). Individuals in the PLUS group who were most likely to improve had better baseline endurance (longer six-minute walk distance) and were more active (greater activity counts by actigraphy) than those who did not improve. Both interventions improved gait speed. Faster walkers and those with greater capacity (i.e., endurance and activity level) may need the challenge of the timing and coordination component to become motor experts in walking and sustain treatment benefits.

